# Engineering *Bacillus subtilis* J46 for efficient utilization of galactose through adaptive laboratory evolution

**DOI:** 10.1186/s13568-024-01666-8

**Published:** 2024-01-29

**Authors:** Jae Woong Choi, Nho-Eul Song, Sang-pil Hong, Young Kyoung Rhee, Hee-Do Hong, Chang-Won Cho

**Affiliations:** Research Group of Traditional Food, Korea Food Research Institute, 245, Nongsaengmyeong-ro, Iseo-myeon, Wanju-gun, 55365 Republic of Korea

**Keywords:** *Bacillus subtilis*, Adaptive laboratory evolution, Galactose, *Leloir* pathway, Reverse engineering

## Abstract

Efficient utilization of galactose by microorganisms can lead to the production of valuable bio-products and improved metabolic processes. While *Bacillus subtilis* has inherent pathways for galactose metabolism, there is potential for enhancement via evolutionary strategies. This study aimed to boost galactose utilization in *B. subtilis* using adaptive laboratory evolution (ALE) and to elucidate the genetic and metabolic changes underlying the observed enhancements. The strains of *B. subtilis* underwent multiple rounds of adaptive laboratory evolution (approximately 5000 generations) in an environment that favored the use of galactose. This process resulted in an enhanced specific growth rate of 0.319 ± 0.005 h^−1^, a significant increase from the 0.03 ± 0.008 h^−1^ observed in the wild-type strains. Upon selecting the evolved strain BSGA14, a comprehensive whole-genome sequencing revealed the presence of 63 single nucleotide polymorphisms (SNPs). Two of them, located in the coding sequences of the genes *araR* and *glcR,* were found to be the advantageous mutations after reverse engineering. The strain with these two accumulated mutations, BSGALE4, exhibited similar specific growth rate on galactose to the evolved strain BSGA14 (0.296 ± 0.01 h^−1^). Furthermore, evolved strain showed higher productivity of protease and β-galactosidase in mock soybean biomass medium. ALE proved to be a potent tool for enhancing galactose metabolism in *B. subtilis*. The findings offer valuable insights into the potential of evolutionary strategies in microbial engineering and pave the way for industrial applications harnessing enhanced galactose conversion.

**Supplementary Information:**

The online version contains supplementary material available at 10.1186/s13568-024-01666-8.

## Key Points

- Following adaptive laboratory evolution in a minimal medium with galactose serving as the selective pressure, the *B. subtilis* BSGA14 strain was successfully developed, exhibiting a specific growth rate of 0.319 ± 0.005 h^−1^.
- Among SNPs after resequencing, mutation in the *araR* gene enhances the uptake of galactose in *Bacillus subtilis*, leading to increased galactose utilization.
- The nonsense mutation in the *glcR* gene, which is involved in carbon catabolite repression (CCR) and sugar phosphorylation, influences galactose metabolism in *B. subtilis*.

**Supplementary Information:**

The online version contains supplementary material available at 10.1186/s13568-024-01666-8.

## Introduction

The gram-positive, rod-shaped bacterium, *Bacillus subtilis* with a tractable genetic code and a well-known metabolic route, has become a key player in the field of microbial engineering. *B. subtilis* serves as a pivotal catalyst in the production of many enzymes such as protease, amylase, lipase, among others, attributed to its prolific secretory protein output (Cui et al. 2018; Schallmey et al. 2004; Ÿztürk et al. 2016). It is renowned for synthesizing a wide spectrum of secondary metabolites, encompassing antibacterial and antifungal compounds (Harwood et al. 2018). Additionally, *B. subtilis* is used in the fermentation of various foods like doenjang, cheonggukjang, natto, and beyond, being recognized as a Generally Recognized As Safe (GRAS) strain (Kimura and Yokoyama 2019). Given the variety of uses for *B. subtilis*, the majority of the biomass is soybean-based biomass (Gopikrishna et al. 2021; Sanjukta & Rai 2016). The utilization of soybean-based biomass not only epitomizes a stride towards sustainable industrial bioprocessing but also provides a fertile ground to delve into the metabolic interactions between *B. subtilis* and the biomass constituents.

A notable constituent of soybean biomass is galactose, a monosaccharide that serves as a significant carbon source (Karr-Lilienthal et al. 2005). Moreover, galactose is frequently present in marine biomass such as macroalgae and is also a principal sugar in dairy by-products. (Chen et al. 2021; Lee et al. 2011; Lim et al. 2013). While a handful of studies have ventured into elucidating galactose utilization or engineering the metabolic pathway in *B. subtilis*, the utilization of galactose utilization from soybean-based biomass by *B. subtilis* remains largely uncharted (Chai et al. 2012; Kim et al. 2010; Krispin and Allmansberger 1998a). Efficient harnessing of galactose can pave the way for amplifying the metabolic efficiency of *B. subtilis* fermentation processes, thereby augmenting the yield of target products.

In the field of microbial engineering, adaptive laboratory evolution (ALE) is a key technique that provides a window through which the innate and potential metabolic capacity of microorganisms can be understood and improved (Mans et al. 2018; Mohamed et al. 2017; Sandberg et al. 2019; Zhang et al. 2022). ALE promotes the natural evolution of desirable features by subjecting microbial populations to predetermined selective pressures over several generations. This allows for the fine-tuning of microbial phenotypes towards certain biotechnological goals. Engineering endeavors involving *B. subtilis* via ALE have predominantly centered around enhancing cell growth rates under varying conditions. Notable studies have explored growth under glucose as the primary carbon source (Liu et al. 2020; Yuan et al. 2019), while others have delved into growth on xylose as the sole carbon substrate (Averesch and Rothschild 2019; Zhang et al. 2015). Additionally, investigations have been carried out with NH_4_Cl serving as the exclusive nitrogen source (Li et al. 2023), and within the milieu of lignocellulosic hydrolysate (Driessen et al. 2023). Through these varied explorations, ALE has emerged as a powerful tool in fine-tuning the metabolic and growth attributes of *B. subtilis* to adapt to distinct nutrient and environmental regimes.

In this study, we employed ALE as a strategic approach to optimize the galactose utilization pathway in *B. subtilis*. Recognizing the significance of galactose as a pivotal carbon source from soybeans and its potential as an energy source for the bacterium, our research aimed to harness the power of evolutionary techniques to enhance metabolic efficiency. By subjecting *B. subtilis* to controlled environmental pressures with galactose as the primary carbon source in minimal medium, we endeavored to unravel the genetic and phenotypic changes that arise, facilitating improved galactose conversion. Our findings not only provide insights into the adaptability and metabolic versatility of *B. subtilis* but also lay the groundwork for future microbial engineering efforts aimed at sustainable bioprocessing using soybean-based biomass.

## Materials and methods

### Bacterial strains, media, and culture conditions

Strains used in this study are listed in Table 1. The strain *B. subtilis* J46 (KCCM12388P) was used as the parental strain for ALE. *Escherichia coli* DH5α and HST04 were employed for plasmid construction and replication. All strains were stored at − 80 °C and revitalized by cultivating at 37 °C in *Luria–Bertani* medium (LB, BD, USA) supplemented with appropriate antibiotics. To select transformants and maintain plasmid, 100 µg/mL ampicillin (Amp) was added for *E. coli.* Also, 5 µg/mL chloramphenicol (Cm) and 10 µg/mL kanamycin (Km) were applied for *B. subtilis*. All antibiotics were purchased from Sigma-Aldrich (USA).

**Table 1:** Bacterial strains and plasmid used in this study

Strain	Characteristic	Sources
*E. coli*		
DH5α	F^–^ φ80*lac*ZΔM15 Δ(*lac*ZYA*arg*F)U169 *rec*A1 *end*A1 *hsd*R17(r_K_^–^, m_K_^+^) *pho*A *sup*E44 λ^–^*thi*-1 *gyr*A96 *rel*A1; Cloning host	Invitrogen
HST04	F^−^, *ara*, Δ(*lac*-*proAB*)[Φ80d*lacZ*ΔM15], *rpsL*(*str*), *thi*, Δ(*mrr*-*hsdRMS*-*mcrBC*), Δ*mcrA*, *dam*, *dcm*	Takara
*B. subtilis*		
J46	KCCM12388P	This study
BSGA14	*B. subtilis* J46 adaptively evolved in M9 medium with galactose as a sole carbon source, KACC92527P	This study
BSGALE1	J46 *araR*^H226R^	This study
BSGALE2	J46 Δ* araR*	This study
BSGALE3	J46 Δ* glcR*	This study
BSGALE4	J46 *araR*^H226R^; Δ* glcR*	This study
*Plasmids*		
pHTCpf1	pHT01 derivative plasmid, containing P*grac*-Cpf1, *Amp*^*R*^, *Km*^R^	Addgene
pADsacA	pAD123 derivative, containing *sacA* targeting 23 bp crRNA transcription module and 1.2 kb donor DNA, *Amp*^*R*^, *Cm*^R^	Addgene
pAD-araR	pADsacA derivative, *araR* cRNA, *Amp*^*R*^, *Cm*^R^	This study
pAD-araRHR	pAD-araR derivative, *araR* cRNA, homologous arm of *araR*, *Amp*^*R*^, *Cm*^R^	This study
pAD-araRdelHR	pAD-araR derivative, *araR* cRNA, homologous arm of *araR* for deletion, *Amp*^*R*^, *Cm*^R^	This study
pAD-glcR	pADsacA derivative, *glcR* cRNA, *Amp*^*R*^, *Cm*^R^	This study
pAD-glcRHR	pAD-glcRHR derivative, *glcR* cRNA, homologous arm of *araR*, *Amp*^*R*^, *Cm*^R^	This study

### Adaptive laboratory evolution and strain isolation

For the ALE experiment, overnight cultivated *B. subtilis* J46 was transferred to 50 mL of M9 minimal medium containing 2% (*w/v*) galactose in 250 mL Erlenmeyer flat-bottom flask at 37 °C with 200 rpm in triplicate (Harwood 1990). Initially, 0.11 percent yeast extract was added to the minimal medium to boost the growth of J46. By progressively lowering the supplementation, growth without supplementation was eventually attained. Batch cultures were manually transferred to fresh medium every 24 h. After cultivation, the optical cell density was measured at 600_ nm_ (OD_600_), and the initial optical density at 600_ nm_ (OD_600_) was set to 0.05. After ALE, single clones were isolated on the M9 galactose agar medium.

### Analytical methods

Cell growth was measured at 600 nm by spectrophotometer (Eppendorf, Germany). Cell culture samples were centrifuged at 13,000 rpm for 10 min, and the supernatant was then conserved for additional research. The concentration of galactose was determined using an HPLC system (Jasco, France) consisting a PU-2089 pump, AS-2057 auto-injector, and RI-2031 differential refractive index (RI) detector (JASCO, France). Aminex HPX 87-H column (Bio-Rad, Richmond, USA) was equipped and 5 mM H_2_SO_4_ (0.5 mL/min) was used as the mobile phase at 60 °C.

### Genomic DNA extraction and whole-genome resequencing, mutation screening

The isolates' genomic DNA was extracted using the MagAttract HMW DNA kit (Qiagen, Germany), following the guidelines provided by the manufacturer. The DNA's concentration was measured with the Qubit 2.0 fluorometer (Invitrogen, USA). To ensure there was no contamination in the DNA or the cultured sample, the 16S rRNA gene was sequenced using the ABI 3730 DNA sequencer (Applied Biosystems, USA). Details regarding the DNA sample's quantity and quality are provided below.

Libraries were produced by fragmenting the genomic DNA to approximately 550 bp using the M220 Focused-ultrasonicator™ (Covaris Ltd, UK). The fragmented DNA's size was then assessed using the Bioanalyzer 2100 (Agilent, USA) and the DNA 7500 kit. The TruSeq DNA Library LT kit (Illumina, USA) was employed to assemble the library, adhering to the manufacturer's guidelines. Comprehensive genome sequencing was executed on the Illumina MiSeq platform, generating 2 × 300 bp paired-end sequences with the 600 cycle (MiSeq Reagent Kit v3) sequencing set.

Sequencing data from Illumina were compiled using SPAdes 3.13.0 (Bankevich and Nurk 2012). The EzBioCloud genome database was employed for the gene identification and functional annotation of the entire genome assembly (Yoon et al. 2017). The prediction of protein-coding sequences (CDSs) was carried out using Prodigal 2.6.2, as described (Hyatt et al. 2010). The CDSs were categorized based on their functions, referencing the orthologous groups from EggNOG 4.5 (accessible at http://eggnogdb.embl.de) (Powell et al. 2012). For a comprehensive functional annotation, the anticipated CDSs were cross-referenced with databases such as Swissprot (Berman et al. 2000), KEGG (Kanehisa et al. 2016), and SEED (Overbeek et al. 2005), utilizing the UBLAST software (Edgar 2010). For screening mutations, a comparative genomic analysis was conducted using CJ Bioscience’s comparative genomics tool.

### Construction of plasmids and mutant strains

All primers used for construction plasmids in this study are listed in Table 2. All the plasmids constructed in this study are detailed in Table 1 and were constructed by Gibson assembly kit (Takara, Japan). To introduce SNP mutations on the J46 genome, a CRISPR-Cpf1-based tool kit was applied (Hao et al. 2020). Single guide RNA and homologous arms were cloned into pADsacA plasmids. A 21-bp gRNA was screened adjacent to the mutation site of the *araR* and *glcR* genes. To yield pAD-araR plasmid, pADsacA was amplified by PCR reaction with primer pairs, araR_crRNA_F, and araR_crRNA_R, then PCR product was used for Gibson assembly. Utilizing the J46 genome as a template, the homologous arms of *araR* were amplified to create pAD-araRHR using araR HR_A_F, araR_HR_A_R, araR_HR_B_F, and araR_HR_B_R. The other plasmids were cloned as described above. Transformation of *E. coli* was performed using recombinant plasmids as described (Inoue et al. 1990). Recombinant *B. subtilis* strains were constructed via the natural transformation method (Chang and Cohen 1979).

**Table 2:** Oligonucleotides used in this study

Primer name	Sequences (5′ to 3′)
araR_crRNA_F	TAAACAAACAATTAACCCCGTATTTCAAATAAAACGAAAGGCTCAGTCGAAAG
araR_crRNA_R	ATACGGGGTTAATTGTTTGTTTACACGCTCCCGGTGCGCCTGTAACATTTATTGTACAACACGA
araR_HR_A_F	TAGGCGTATCACGAGGCCCTTTCGTCGTGCAAGGCGGAGGCACCTTTGT
araR_HR_A_R	CGGATGAACGGATTTATACAGGCGCGCCGGGAGCGTGAGTTGTTTCCTTC
araR_HR_B_F	GAAGGAAACAACTCACGCTCCCGGCGCGCCTGTATAAATCCGTTCATCCG
araR_HR_B_R	TTTGTATTGTGTTGAAATATGTTTTGTTATTCATTCAGTTTTCGTGCGGACTG
araR_HR_del_A_F	AATAGGCGTATCACGAGGCCCTTTCGTATGTTACCAAAATACGCGCAAGTAAA
araR_HR_del_A_R	TCCGTTGTAAATGTCACGATCATATCCGGAGGCAAAAGGAATGCCGTTTTTCTCC
araR_HR_del_B_F	GGAGAAAAACGGCATTCCTTTTGCCTCCGGATATGATCGTGACATTTACAACGGA
araR_HR_del_B_R	TTTGTATTGTGTTGAAATATGTTTTGTTATTCATTCAGTTTTCGTGCGGACTG
glcR_crRNA_F	CGCTTCACCATGCCATCCTCTATTTCAAATAAAACGAAAGGCTCAGTCGAAAG
glcR_crRNA_R	AGAGGATGGCATGGTGAAGCGCACGCTCCCGGTGCGCCTGTAACATTTATTGTACAACACGA
glcR _HR_A_F	TAGGCGTATCACGAGGCCCTTTCGTAATTACAACGGAGCAGATCTGTACG
glcR _HR_A_R	TTTTCCGCTTCACCATGCCATCCTCTTCATGCGCAAATTGTAATGCCATG
glcR _HR_B_F	CATGGCATTACAATTTGCGCATGAAGAGGATGGCATGGTGAAGCGGAAAA
glcR _HR_B_R	TTGTATTGTGTTGAAATATGTTTTGTCAGTCCTTTCCTTCATCCTGCTC
sigA_RT_F	GGCAGAGAACCAACACCTGA
sigA_RT_R	TCACCAAGGTGCGAGTCATC
araE_RT_F	CTTTGTCGCTTCACGCTCTG
araE_RT_R	TCCTCTGATGATGCTCGGGA
araR_RT_F	CTTTGTCGCTTCACGCTCTG
araR_RT_R	TCCTCTGATGATGCTCGGGA
araB_RT_F	TTGGCATTACAGAGCCAGGG
araB_RT_R	CGGGAGAATTCCGTTGTCCA
araD_RT_F	CCTTGATGGAGAGGTCGTCG
araD_RT_R	AGCTTGTCGCCCATTGAGAA
araL_RT_F	ATGATTGGGGCGATAGAGGC
araL_RT_R	ATGTGCGGACAGTCCCATTG
araM_RT_F	CTTGACGCCTGTATCGCACA
araM_RT_R	AAATATGATGCTCGCCGCCT
araN_RT_F	TTGTGCCGAAACAAGCCAAG
araN_RT_R	CTCCAGACATCCCAGCGAAG
abnA_RT_F	TCGGGCAATGACCAATGGAA
abnA_RT_R	AGCCCGAGCTCCAATTCAAA
galE_RT_F	TCCGACAAAAGACGGGACAG
galE_RT_R	TGTGCCTGTTCCAAGGTTGT
galT_RT_F	AGTGAAGTGGCCGATGTCTG
galT_RT_R	ATTATGGGGCGTATCGCCTG
galK_RT_F	CTATAACGGCGGGCATGTCT
galK_RT_R	TCCTTGATGCCTGCGTTTCT

### RNA extraction and quantitative real-time polymerase chain reaction (PCR) experiments

Wild type strain *B. subtilis* J46, BSGA14, and mutant strains were cultivated in SP minimal medium with 20 g/L galactose as the sole carbon source. At the exponential growth phase (OD_600_ = 1–2), 5 mL of each flask was sampled by centrifugation at 13,000 rpm for 5 min. Total RNA was isolated using an RNeasy kit (Qiagen, Germany) following the manufacturer's instructions. Sampled RNA was quantified using a Qubit dsDNA HS Assay Kit (Thermo Fisher Scientific, USA) with a Qubit 2.0 fluorometer (Thermo). For cDNA synthesis, 2 μg of RNA was reverse transcribed using the PrimeScript™ kit (TAKRA, Japan) with gDNase and random primers. The quantitative RT-PCR was performed using the StepOnePlus RT-PCR system (Applied Biosystems, USA) and TB Green® Premix Ex Taq™ II (TAKARA) according to the manufacturer's instructions. All primers were designed by Primer-BLAST (Ye et al. 2012), specific binding was confirmed in the *B. subtilis* J46 genome and the primer sequences are listed in Table 2. Transcription levels were normalized to that of the *sigA* gene.

### Assay of protease and β-galactosidase activity

Wild type and mutant strains were inoculated from stocks from a deep freezer at − 80 °C in LB medium and overnight (16 h, 37 °C). Cells were transferred into mock soybean biomass medium (50 g/L soytone, 5 g/L glucose, and 20 g/L galactose) and incubated over 24 h at 37 °C. Cultures were centrifuged at 13,000 rpm for10 min at 4 °C, with the supernatant being used for further investigation and the pellet being discarded. Protease kit (Thermo) and β-galactosidase assay kit from (Abcam, USA) were utilized by the manufacturer's instructions.

## Results

### Adaptive laboratory evolution of *B. subtilis* J46 for galactose utilization in a minimal medium

In a recent study, we identified *B. subtilis* J46, isolated from fermented soybeans, as a strain that synthesizes benzaldehyde, trimethylpyrazine, and 3-hydroxy-2-butanone at significant levels (Hong et al. 2019). To elevate productivity, a fast-growing strain is needed under major monosaccharide conditions in soybean biomass. In soybean dry biomass, galacto-oligosaccharides, which include raffinose, stachyose, and verbascose, constitute roughly 5%, whereas the starch content is less than 1% (Karr-Lilienthal et al. 2005). Numerous monosaccharides, such as glucose, galactose, sucrose, arabinose, glycerol, etc., can be utilized by *B. subtilis* (Fisher and Sonenshein 1991). However, there are few studies to engineer *B. subtilis* for an efficient metabolic pathway for galactose (Singh et al. 2017). In order to engineer *B. subtilis* for increased fitness when galactose is the only carbon source, we performed ALE.

**Fig. 1:**
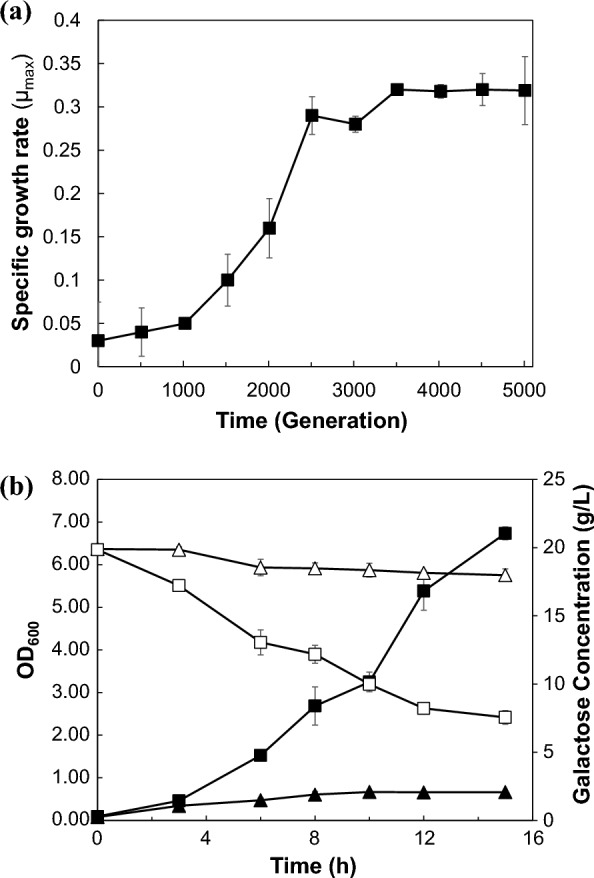
Adaptive laboratory evolution of *B. subtilis* J46. **a** Change in specific growth rate (μ_max_) as a function of accumulated generations of *B. subtilis* J46 adaptively evolved in M9 minimal medium supplemented 2% (*w/v*) galactose. **b** Cell growth profile of parental strain (J46) and evolved strain (BSGA14) under M9 minimal medium containing 2% (*w/v*) galactose. Black and white symbols mean optical density at 600 nm and galactose concentration (g/L), respectively. Triangles and squares represent samples from *B. subtilis* J46 and BSGA14, respectively. The standard deviation estimated from triplets is represented by the error bars

To promote the growth of *B. subtilis* J46 and support a larger cell population, 1% yeast extract was initially added to M9 minimal medium containing 2% (w/v) galactose. After 1000 generations, cultivation was carried out without the addition of yeast extract. Cell stocks were systematically preserved at − 80 °C in a deep freezer every 500 generations, to facilitate ongoing analysis and inoculation procedures. Changes in the growth profile during ALE are presented in Fig. 1a. Specific growth rate at the end-point population was 0.319 ± 0.005 h^−1^, improving tenfold faster than the parental strain (0.03 ± 0.008 h^−1^). The significant increase in fitness on galactose was confirmed between 1000 and 2500 generations of ALE. Single colonies were isolated from the last evolved population and analyzed for growth and galactose profile, as well as for further downstream analysis (Additional file 1: Fig. S1). Finally, the fastest-growing strain was named BSGA14 (deposited in KACC 92528P). Compared to the wild-type strain, which achieved an optical density (OD_600_) of 0.67 after 16 h of cultivation, the optical density of BSGA14 reached 6.96. This significant increase demonstrates the enhanced galactose consumption ability of BSGA14 (Fig. 1b).

### Genomic resequencing of strains BSGA14

**Fig. 2:**
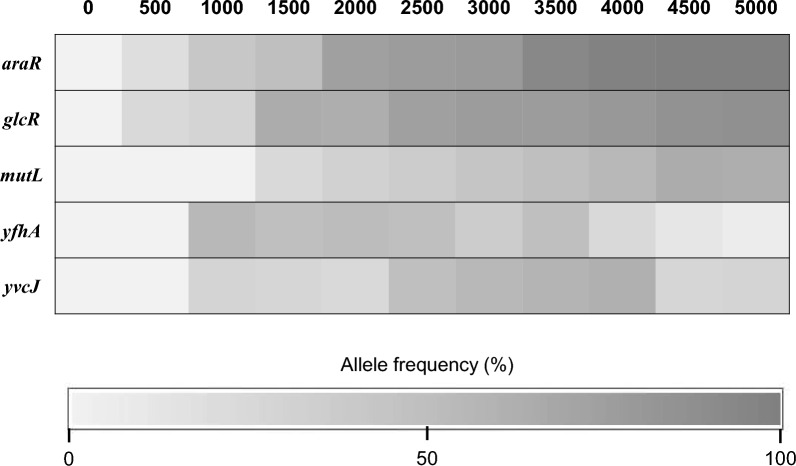
Frequencies of mutations at a given generation during ALE. By dividing the count of mutations by the coverage at this site, the frequency of mutations was calculated

Uncovering significant mutations that enhance cell growth under selective pressure is a pivotal aspect of ALE (Driessen et al. 2023; Kang et al. 2019; Kim et al. 2022). The technique of reverse engineering is employed to discern causative mutations from incidental ones. Key mutations were pinpointed through whole-genome resequencing of the BSGA14 strain, using its parental strain as a benchmark. A total of 63 SNP mutations were identified in the BSGA14 strain (Additional file 1: Table S1). Out of these, two SNPs, *araR*^H226R^ and *glcR*^243InsA^, were singled out as notable alterations (Table 3). Our attention was centered on transcriptional factors within the carbon catabolic pathway, given the significant evolutionary impact attributed to transcription factors like repressors (Igler et al. 2018; Ottilie et al. 2022). While no mutations were observed in the galactose metabolic pathway, it's apparent that the carbon catabolic repressor can influence various monosaccharides, including galactose, beyond just arabinose or glucose. To scrutinize the temporal emergence of mutations during ALE, the frequency of mutations was evaluated at various intervals (Fig. 2, Additional file 2: Table S2). Three arbitrarily chosen mutations (*mutL*, *yfhA*, and *yvcJ*) in BSGA14 were noted to have low frequencies (below 40%) in the final population, with no discernible trends. Conversely, the frequencies of *araR*^H226R^ and *glcR*^243InsA^ mutations surged from 0 to over 90%. Notably, a significant increment was recorded between the 1000 to 2000 generation span, which aligned with the escalation of the specific growth rate.

**Table 3:** Key mutations in BSGA14 strain

Locus	Gene	Nucleotide change	Amino acid exchange
1220431	*araR*, Arabinose metabolism transcriptional repressor	677 A > G	H226R
1474174	*glcR*, HTH-type transcriptional repressor	243InsA	Non-sense mutation

### Mutation on *araR* increases galactose utilization via enhancing galactose uptake

The gene *araR*, pivotal in *Bacillus subtilis*, encodes for the transcriptional repressor governing arabinose metabolism. It has been previously established that *araR* plays a cardinal role in the regulation of the *araABDLMNPQ-abfA* metabolic operon, serving as an integral controller in the metabolic pathway (Mota et al. 1999; Zhang et al. 2015). In addition, *araR* controls the expression of *araE* which is involved in the degradation and transport of arabinose-containing polysaccharides, xylose, and galactose, as well as intracellular catabolism of arabinose and arabinose oligomers. It is reported that *araE* has broad substrate specificity, not only arabinose but also galactose and xylose (Krispin & Allmansberger 1998b). So single amino acid substitution in *araR* could inhibit DNA binding and increase expression of *araE*. *araR*^H226R^ mutation was studied and demonstrated a lower repression level compared to wild type *araR* (Franco et al. 2006). To elucidate the significant mutation effect on galactose metabolism, single mutants of *araR*^H226R^, BSGALE1 was constructed utilizing the CRISPR-cpf1-mediated reverse engineering approach.

**Fig. 3:**
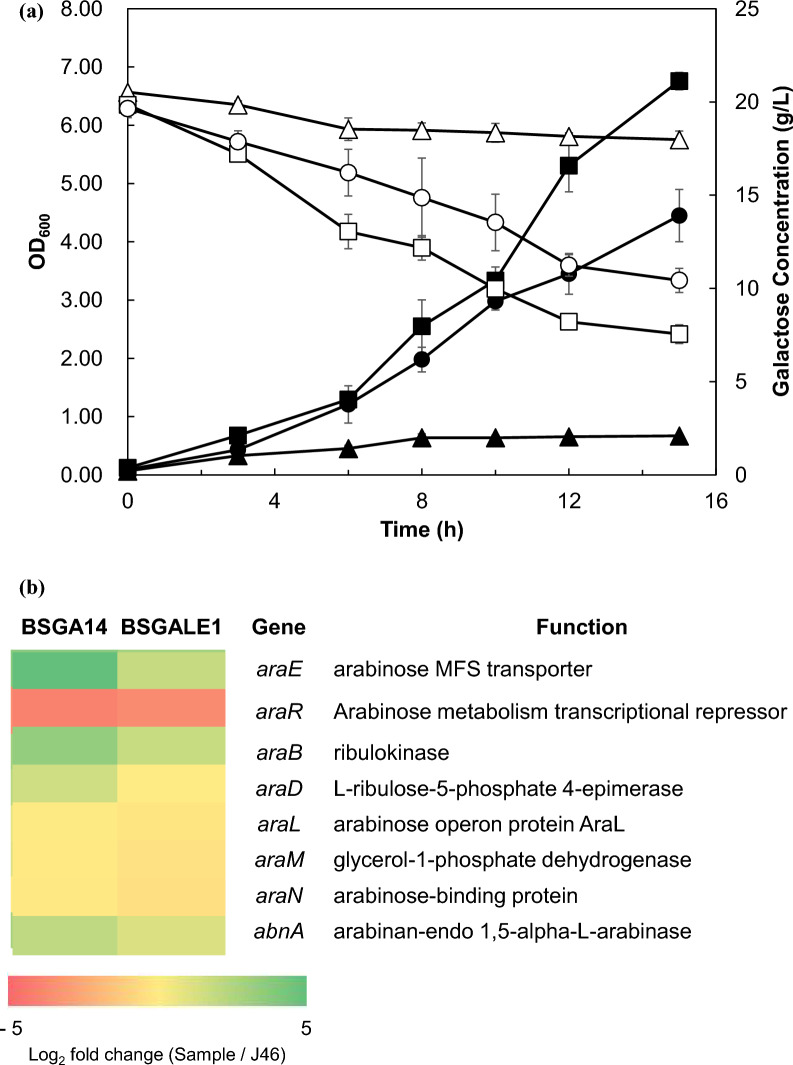
Reverse engineering of *B. subtilis* J46 introduced mutation of *araR*^H226R^. **a** The growth profile and galactose consumption of *B. subtilis* J46, BSGA14, and BSGALE1 in M9 minimal medium containing 2% (*w/v*) galactose. Black and white symbols mean optical density at 600 nm and galactose concentration (g/L), respectively. Triangles, squares, and circles represent samples from *B. subtilis* J46, BSGA14, and BSGALE1 respectively. The standard deviation estimated from triplets is represented by the error bars. **b** Change in gene expression levels of the arabinose metabolic pathways in response to galactose adaptation. Log_2_ fold change compared to genes related to arabinose catabolism of the parental strain

As expected, BSGALE1 showed high cell growth density compared to wild type strain in M9 minimal medium with galactose as the sole carbon source (Fig. 3a). The specific growth rate of BSGALE1 was 0.214 ± 0.013 h^−1^. Also, expression levels of the arabinose metabolic pathway including *araE* which is important for galactose uptake were measured (Fig. 3b, Additional file 3: Table S3). The expression of *araE* (increased by 3.99-fold and 2.88-fold), *araB* (increased by 3.45-fol and, 2.87-fold), and *abnA* (increased by 2.99-fold and 2.65-fold) was much higher in BSGA14 and BSGALE1, respectively, compared to wild type strain. Moreover, *araR* was seen to be repressed, most likely as a result of *araR*^H226R^'s impact. The homodimer of araR was recognized to be impacted by the 226th amino acid position (Franco et al. 2006; Procházková et al. 2012). Dimerization may be inhibited by amino exchange. To delve deeper into the impact of the *araR* deletion on the galactose metabolic pathway, *araR* was removed from the J46 strain, resulting in the construction of the BSGALE2 strain. Contrary to expectations, the specific growth rate of BSGALE2 was 0.170 ± 0.009 h^−1^ and it was proven that cell density of BSGALE2 was lower than BSGALE1’s (Additional file 1: Fig. S2). Optimal regulation of the arabinose metabolic pathway enhanced galactose utilization.

### Effect of the nonsense-mutation in *glcR* induces acceleration of phosphosugar catabolic reaction

**Fig. 4:**
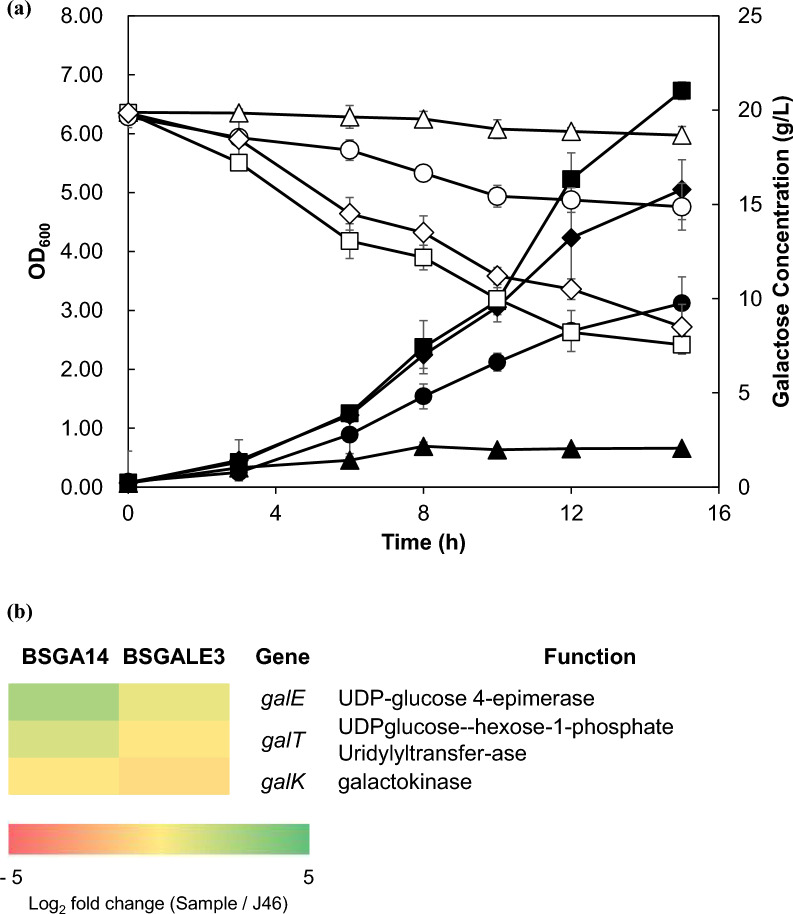
Reverse engineering of *B. subtilis* J46 introduced mutation of Δ*glcR*. **a** The growth profile and galactose consumption of *B. subtilis* J46, BSGA14, BSGALE3 and BSGALE4 in M9 minimal medium containing 2% (*w/v*) galactose. Black and white symbols mean optical density at 600 nm and galactose. Triangles, squares, circles, and diamonds represent samples from *B. subtilis* J46, BSGA14, BSGALE1 and BSGALE4 respectively. The standard deviation estimated from triplets is represented by the error bars. **b** Change in gene expression levels of the galactose metabolic pathways. Log_2_ fold change compared to genes related to galactose catabolism of the parental strain

To date, our understanding suggests that, apart from the missense mutation in the *araR* gene, no other mutations are directly linked to the galactose metabolic pathway. Nonetheless, the *glcR* gene could potentially be an influential mutation target, especially when considering indirect mechanisms. The *glcR* gene plays a crucial role in carbon catabolite repression (CCR), which prioritizes the metabolism of a preferred carbon source (typically glucose) over other alternative sources (Stülke et al. 2001). In *B. subtilis*, galactose is primarily metabolized through the *Leloir* pathway, and the subsequent breakdown of phosphosugars, including glucose 1-phosphate produced from this pathway, is essential for cellular viability (Prasad and Freese 1974). Given that *glcR* is predominantly involved in sugar phosphorylation, its deletion could potentially boost the cell growth rate when cultivated in a galactose minimal medium (Morabbi Heravi et al. 2019). Consequently, the BSGALE3 strain with a deleted *glcR* gene was developed. BSGALE3 showed a higher specific growth rate (0.216 ± 0.012 h^−1^) than that of wild type (Fig. 4a). The relative expression levels of *Leloir* pathway genes were measured. There was a modest upregulation observed in several key genes: *galE* expression increased by 3.46-fold in BSGA14 and 2.98-fold in BSGALE3; *galT* expression rose by 3.12-fold in BSGA14 and 2.64-fold in BSGALE3; and *galK* expression was up by 2.65-fold in BSGA14 and 1.99-fold in BSGALE3 (Fig. 4b, Additional file 4: Table S4).

In the investigation of the cumulative impact of the *araR*^H226R^ and *ΔglcR* mutations, we established the BSGALE4 strain. Upon measurement, the specific growth rate was determined to be 0.296 ± 0.01 h^−1^ (Fig. 4a). These findings underscore the significance of these two mutations in the evolution of the strain.

### Soybean broth utilization in BSGA14 and BSGALE4 strain

**Fig. 5:**
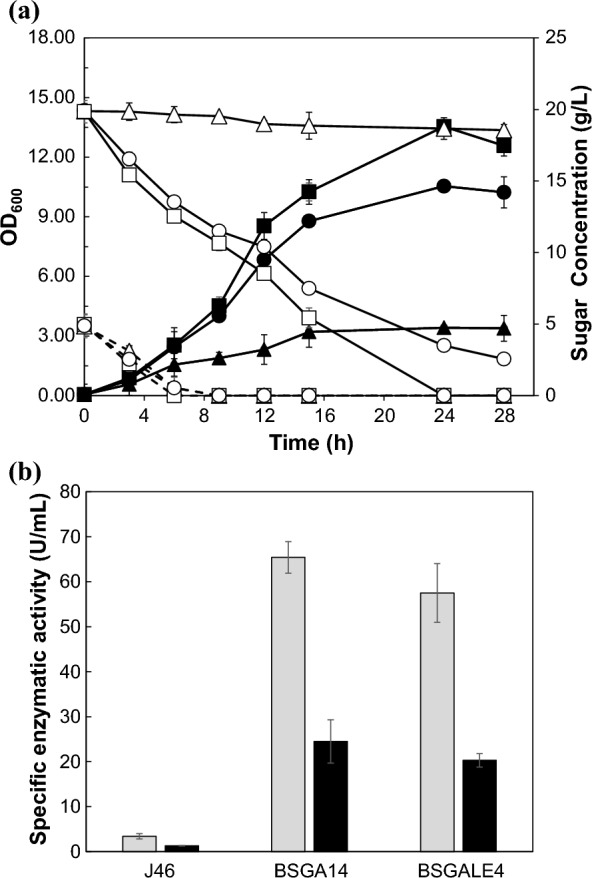
Cultivation in mock soybean hydrolysis medium. **a** The growth profile and sugar consumption of *B. subtilis* J46, BSGA14, and BSGALE4. Black and white symbols mean optical density at 600 nm and monosaccharides. Triangles, squares, circles and diamonds represent samples from *B. subtilis* J46, BSGA14, BSGALE1 and BSGALE4 respectively. Solid and dashed lines mean galactose and glucose concentration, respectively. **b** Enzyme activity from *B. subtilis* J46, BSGA14, and BSGALE4 after cultivation. Grey and black mean protease and β-galactosidase activity, respectively

The strains J46, BSGA14, and BSGALE4 were cultivated on a simulated soybean biomass medium, enriched with galactose as a primary monosaccharide, aiming to broaden the applicability of the developed strains. As depicted in Fig. 5, the J46 strain exhibited incomplete galactose consumption, with cell growth registering below an optical density (OD_600_) of 4. Conversely, both the evolved and engineered strains demonstrated enhanced cell density, surpassing an OD_600_ of 10. The biomass of BSGA14 showed 5.47-fold higher in 24 h than that of J46. In addition, the reverse engineered strain BSGALE4 had 3.08-fold biomass than the J46 in 24 h. A near uniform utilization of glucose and galactose was observed. Furthermore, a significant enhancement in enzymatic activity was evident, corresponding to the increased cell densities. Specifically, the BSGALE4 strain manifested a protease activity of 20.5 ± 4.8 U/mL and a β-galactosidase activity of 65.4 ± 3.5 U/mL. In comparison, the BSGA14 strain exhibited protease activity at 65.4 ± 6.5 U/mL and β-galactosidase activity at 20.3 ± 1.5 U/mL. Markedly, the wild type strain J46 demonstrated substantially lower enzyme activities, with protease and β-galactosidase activities recorded at 3.4 ± 0.6 U/mL and 1.3 ± 0.05 U/mL, respectively. Contrasting with the wild type, the evolved and engineered strains displayed enhanced enzyme activities, underscoring their improved efficiency in galactose utilization and superior metabolic capabilities.

## Discussion

The adaptability and resilience of microbial life are demonstrated by the ability of microorganisms to adjust to shifting environmental conditions and selective forces. In our pursuit to harness this adaptability for biotechnological applications, we employed ALE to improve the galactose utilization capability of *B. subtilis*. There were several trials to engineer *B. subtilis* with ALE (Driessen et al. 2023; Li et al. 2023; Zeigler and Nicholson 2017). Zhang et al. have reported that *B. subtilis* was adapted under xylose as a selection pressure and validated several key mutations (Zhang et al. 2015). ALE was utilized not only to address carbon stress but also to promote rapid development, efficient use of nitrogen sources, and tolerance to lignocellulosic hydrolysate (Driessen et al. 2023; Li et al. 2023; Liu et al. 2020).

This study discovered *araR* and *glcR* mutations, which were responsible for more than 80% of the fitness improvements of BSGA14 over the J46 strain. All mutations correlated with transcription factors on carbon metabolic pathway. Transcription factors are often reported as the causal mutations in various scenarios. This identification is based on changes in their affinity for binding to target operon sequences, alterations in their interactions with other cellular components, or shifts in their capability to form dimer, trimer, or tetramer structures (Ottilie et al. 2022; Phaneuf et al. 2020). *araR* as a GntR family member regulates of carbon catabolism not only of *B. subtilis* but also other microorganisms (Correia et al. 2014; Kuge et al. 2015). Low DNA binding affinity to the arabinose operon was caused by a non-synonymous nucleotide substitution location in *araR*^H226R^ close to the arabinose binding site on the C-terminal domain (Franco et al. 2006). The *araR*-regulated proton symport-type permease *araE* has a wide range of substrate specificity, encompassing arabinose, xylose, and galactose. (Ferreira and Sá-Nogueira 2010; Krispin and Allmansberger 1998b). Due to the *araR*^H226R^ mutation, *araE* was highly expressed, which improved galactose uptake into the cell. The *araR* deleted strain BSGALE2 demonstrated a lower specific growth rate than the *araR*^H226R^ mutant, which was unexpected (Additional file 1: Fig. S2). Additionally, our research aligns with previous findings, such as those by Hernández-Montalvo et al. (2003) and Lu et al. (2012), which demonstrated increased metabolite productivity and cell density following modifications in galactose transporter expression (Hernández-Montalvo et al. 2003; Lu et al. 2012). Considering these insights, finely adjusting the expression level of *araE* presents a promising approach for engineering *B. subtilis* for galactose utilization.

To the best of our knowledge, there is no established direct link between the *glcR* gene and the galactose metabolic pathway, according to current research findings. *glcR* is predominantly involved in carbon catabolite repression, a crucial regulatory mechanism. This process suppresses the metabolism of secondary carbon sources when glucose is present in the cytoplasm (Stülke et al. 2001). In a recent work, *glcR* deleted *B. subtlis* was created, and in comparison to the parent strain, 314 genes were elevated and 195 genes were downregulated by at least two-fold (Niu et al. 2021). These changes reflect the complex network of gene regulation influenced by *glcR*, particularly in the context of phosphosugar stress response and cellular metabolism. Intriguingly, our observations suggest that even in glucose-absent conditions, like the minimal medium used in ALE, the basal repression commonly attributed to *glcR* might be mitigated by its deletion. This hypothesis hints at a complex, possibly indirect interaction within the metabolic network. A deeper exploration into this network, particularly focusing on how *glcR* deletion affects the galactose metabolic pathway, is essential for a more comprehensive understanding of these interactions.

The high productivity of protease (20.5 ± 4.8 U/mL) and β-galactosidase (65.4 ± 3.5 U/mL) in the evolved strain BSGA14, owing to its high cell density, underscores the possible industrial uses of our discoveries. (Fig. 5). This study suggests that modified *B. subtilis* may be used for galactose-rich biomass fermentation, such as that of dairy waste and microalgae. (Sar et al. 2022; Tang et al. 2020; Wu et al. 2015). Sahoo et al. reported on the production of nattokinase through the use of cheese whey as a low-cost fermentation medium (Sahoo et al. 2020). They presented a thorough cost–benefit analysis of producing enzymes from waste streams. Given the high lactose content of cheese whey, this research may help increase enzyme productivity.

### Supplementary Information

**Additional file 1: Table S1**. Mutations of *Bacillus subtilis* BSGA14 strain. **Figure S1.** Isolation of engineered strain via adaptive laboratory evolution using galactose as a sole carbon source. The specific growth rate was measured and the number 14 strain was designated as BSGA14. **Figure S2.** Reverse engineering of *B. subtilis* J46 introduced mutation of *araR*^H226R^ and Δ*araR* (a) The growth profile and galactose consumption of BSGALE1 and BSGALE2 in M9 minimal medium containing 2% (*w/v*) galactose. Black and white symbols mean optical density at 600 nm and galactose concentration (g/L), respectively. Triangles and diamonds represent samples from BSGALE1 and BSGALE2 respectively. The standard deviation estimated from triplets is represented by the error bars.

**Additional file 2: Table S2**. Frequencies of mutations in each population during adaptive laboratory evolution.

**Additional file 3: Table S3**. Transcriptomic analysis of J46, BSGA14, and BSGALE1 related to arabinose catabolism.

**Additional file 4: Table S4**. Transcriptomic analysis of J46, BSGA14, and BSGALE3 related to galactose catabolism.

## Data Availability

The whole genome sequencing data of wild type strain *B. subtilis* J46 and BSGA14 were deposited at NCBI (https://www.ncbi.nlm.nih.gov/) under BioProject accession number (J46 strain: PRJNA1028132, BSGA14 strain: PRJNA1028133).

## Abbreviations

*E. coli*: *Escherichia coli*
*B. subtilis*: *Bacillus subtilis*
ALE: Adaptive laboratory evolution
