# Supplementary material for: Engineering Bacillus subtilis J46 for efficient utilization of galactose through adaptive laboratory evolution
Source: AMB Express. 2024 Jan 29;14:14. doi: 10.1186/s13568-024-01666-8 (PMC10822834; doi:10.1186/s13568-024-01666-8)
Supplement: Supplementary file 1 — Additional file 1: Table S1. Mutations of Bacillus subtilis BSGA14 strain. Figure S1. Isolation of engineered strain via adaptive laboratory evolution using galactose as a sole carbon source. The specific growth rate was measured and the number 14 strain was designated as BSGA14. Figure S2. Reverse engineering of B. subtilis J46 introduced mutation of araRH226R and ΔaraR (a) The growth profile and galactose consumption of BSGALE1 and BSGALE2 in M9 minimal medium containing 2% (w/v) galactose. Black and white symbols mean optical density at 600 nm and galactose concentration (g/L), respectively. Triangles and diamonds represent samples from BSGALE1 and BSGALE2 respectively. The standard deviation estimated from triplets is represented by the error bars. [file 13568_2024_1666_MOESM1_ESM.docx]

# Additional file

**Enhanced Galactose Utilization in *Bacillus subtilis* via Adaptive Laboratory Evolution**

Jae Woong Choi^1^, Nho-Eul Song^1^, Sang-pil Hong^1^, Young Kyoung Rhee^1^, Hee-Do Hong^1^, and Chang-Won Cho^1^*

^1^ Research Group of Traditional Food, Korea Food Research Institute, 245, Nongsaengmyeong-ro, Iseo-myeon, Wanju-gun 55365, Korea

* Corresponding author:

Chang-Won Cho^1^, Tel: +82-63-219-9312, E-mail: cwcho@kfri.re.kr

## Table S1. Mutations of *Bacillus subtilis* BSGA14 strain

| **Ref. Position** | **Ref. Base** | **Qry. Base** | **Ref. FeatureName** | **Mutation Type** | **SNP Type** | **Gene** |
| --- | --- | --- | --- | --- | --- | --- |
| 46223 | T | C | BSU_22010 | Synonymous | Substitution | DNA-directed DNA polymerase |
| 87567 | C | T | BSU_22410 | R12K | Substitution | Aspartate 1-decarboxylase |
| 89432 | . | T | . | Intergenic | Insertion |  |
| 242186 | . | T | . | Intergenic | Insertion |  |
| 294025 | A | . | . | Non-Synonymous | Deletion | uncharacterized protein |
| 452700 | G | A | BSU_27050 | A106V | Substitution | Cytoplasm; Kinase; Phosphotransferase system; Sugar transport; Transferase; Transport. |
| 453765 | T | C | BSU_27070 | D66G | Substitution | Cytoplasm; Kinase; Phosphotransferase system; Sugar transport; Transferase; Transport. |
| 465022 | C | T | BSU_27140 | P329S | Substitution | Putative peptidoglycan O-acetyltransferase YrhL |
| 606392 | C | T | . | Intergenic | Substitution |  |
| 626536 | G | A | BSU_28680 | R120Q | Substitution | FAD; Flavoprotein; Oxidoreductase. |
| 668251 | G | A | BSU_29090 | Synonymous | Substitution | DNA damage; DNA repair; DNA replication; DNA-binding; DNA-directed DNA polymerase; Exonuclease; Hydrolase; Nuclease; Nucleotidyltransferase; Transferase. |
| 675565 | A | . | . | Intergenic | Deletion |  |
| 681172 | C | T | BSU_29200 | E175K | Substitution | Antibiotic resistance; ATP-binding; Cytoplasm; Fatty acid biosynthesis; Fatty acid metabolism; Ligase; Lipid biosynthesis; Lipid metabolism; Nucleotide-binding. |
| 752525 | T | . | BSU_30000 | Non-Synonymous | Deletion | Cell membrane; Membrane; Transmembrane; Transmembrane helix. |
| 752942 | . | A | BSU_30000 | Non-Synonymous | Insertion | Cell membrane; Membrane; Transmembrane; Transmembrane helix. |
| 765263 | T | G | . | Intergenic | Substitution |  |
| 831129 | G | A | BSU_30800 | Synonymous | Substitution | 1,4-dihydroxy-2-naphthoyl-CoA synthase |
| 857276 | . | C | . | Intergenic | Insertion |  |
| 858417 | C | T | . | Intergenic | Substitution |  |
| 859874 | C | T | . | Intergenic | Substitution |  |
| 859908 | A | G | . | Intergenic | Substitution |  |
| 859990 | G | A | . | Intergenic | Substitution |  |
| 882853 | C | T | BSU_31200 | A448T | Substitution | ATP-binding; Disulfide bond; Kinase; Magnesium; Nucleotide-binding; Rhamnose metabolism; Transferase. |
| 889303 | A | . | . | Intergenic | Deletion |  |
| 920669 | A | G | BSU_31520 | Y143C | Substitution | Histidine kinase |
| 929196 | A | G | BSU_31580 | T447A | Substitution | Na(+)-malate symporter |
| 1141683 | T | . | BSU_33510 | Non-Synonymous | Deletion | Cu(+) exporting ATPase |
| 1168180 | A | . | . | Intergenic | Deletion |  |
| 1172514 | C | T | . | Synonymous | Substitution | hypothetical protein |
| 1220431 | A | G | BSU_33970 | H226R | Substitution | Arabinose metabolism transcriptional repressor |
| 1270572 | C | T | BSU_34390 | A397V | Substitution | Para-nitrobenzyl esterase |
| 1303639 | . | A | BSU_30400 | Non-Synonymous | Insertion | Sensory transduction protein BceR |
| 1307971 | T | C | BSU_34770 | E30G | Substitution | Nucleotide-binding protein YvcJ |
| 1474174 | . | T | BSU_36300 | Non-Synonymous | Insertion | HTH-type transcriptional repressor GlcR |
| 1538617 | A | . | . | Intergenic | Deletion |  |
| 1542967 | C | T | BSU_36760 | C224Y | Substitution | UDP-N-acetylglucosamine 1-carboxyvinyltransferase |
| 1650934 | C | T | BSU_38120 | D44N | Substitution | Rod shape-determining protein RodA |
| 1809288 | T | C | BSU_39510 | K2E | Substitution | Uncharacterized N-acetyltransferase YxeL |
| 1890202 | G | A | BSU_40250 | H119Y | Substitution | Formaldehyde dehydrogenase |
| 1907448 | C | T | BSU_40380 | G22D | Substitution | Two-component system YycFG regulatory protein |
| 1947921 | T | C | BSU_33370 | K665E | Substitution | Formate dehydrogenase |
| 1972167 | T | C | . | Intergenic | Substitution |  |
| 2036440 | . | T | BSU_00660 | Non-Synonymous | Insertion | Non-specific serine/threonine protein kinase |
| 2216241 | T | C |  | Intergenic | Substitution |  |
| 2306909 | A | G | BSU_03230 | Y214C | Substitution | Proline-responsive transcriptional activator PutR |
| 2392663 | . | G | BSU_03810 | Non-Synonymous | Insertion | Uncharacterized ABC transporter permease protein YclO |
| 2410605 | A | G | BSU_03981 | E172G | Substitution | Protein-N(pi)-phosphohistidine--D-mannitol phosphotransferase |
| 2486868 | A | G | BSU_04720 | D125G | Substitution | Non-specific serine/threonine protein kinase |
| 2504043 | A | . | . | Non-Synonymous | Deletion | uncharacterized protein |
| 2545634 | A | . | . | Intergenic | Deletion |  |
| 2587112 | T | . | . | Intergenic | Deletion |  |
| 2616306 | A | G | BSU_05970 | T49A | Substitution | Redox-sensing transcriptional repressor Rex |
| 2706301 | . | T | . | Non-Synonymous | Insertion | hypothetical protein |
| 2832606 | C | T | BSU_00810 | Synonymous | Substitution | Probable tRNA-dihydrouridine synthase |
| 2882228 | A | G | BSU_08460 | H205R | Substitution | Probable siderophore transport system permease protein YfhA |
| 2906994 | C | T | . | Synonymous | Substitution | uncharacterized protein |
| 2983659 | T | C | BSU_09290 | W88R | Substitution | Glycerol kinase |
| 3413566 | T | C | BSU_13560 | I259V | Substitution | S-methyl-5-thioribose kinase |
| 3488491 | . | T | . | Intergenic | Insertion |  |
| 3776519 | C | . | BSU_17050 | Non-Synonymous | Deletion | DNA mismatch repair protein MutL |
| 3817238 | T | . | . | Intergenic | Deletion |  |
| 3926950 | A | . | . | Intergenic | Deletion |  |
| 4001235 | G | A | BSU_19450 | A130T | Substitution | RsbT co-antagonist protein RsbRC |


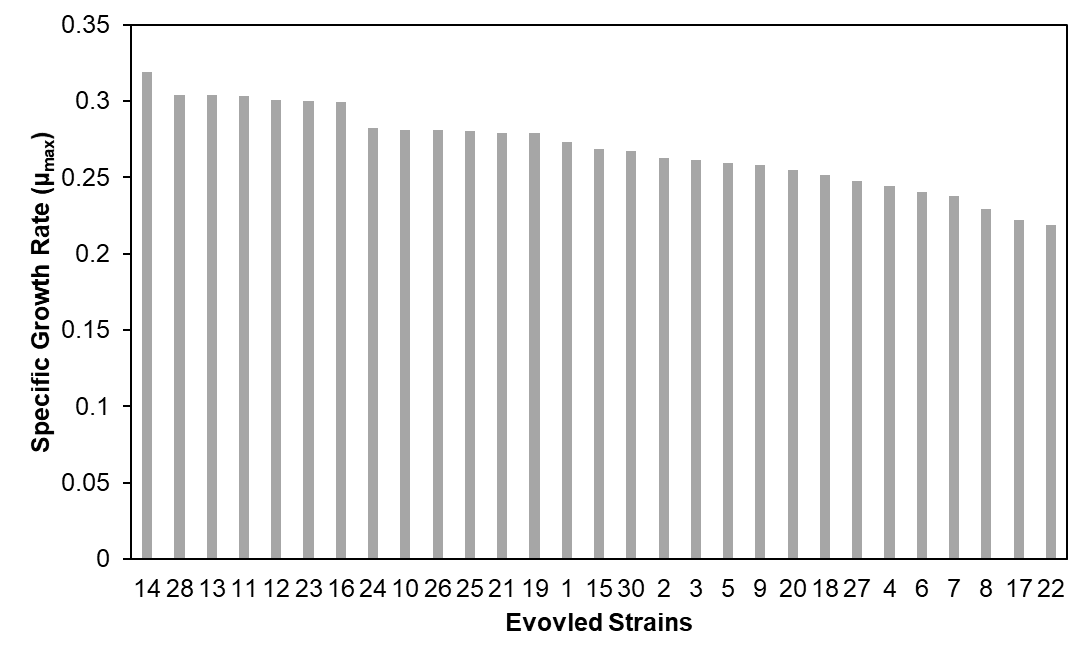


## **Figure S1**. Isolation of engineered strain via adaptive laboratory evolution using galactose as a sole carbon source. The specific growth rate was measured and the number 14 strain was designated as BSGA14.
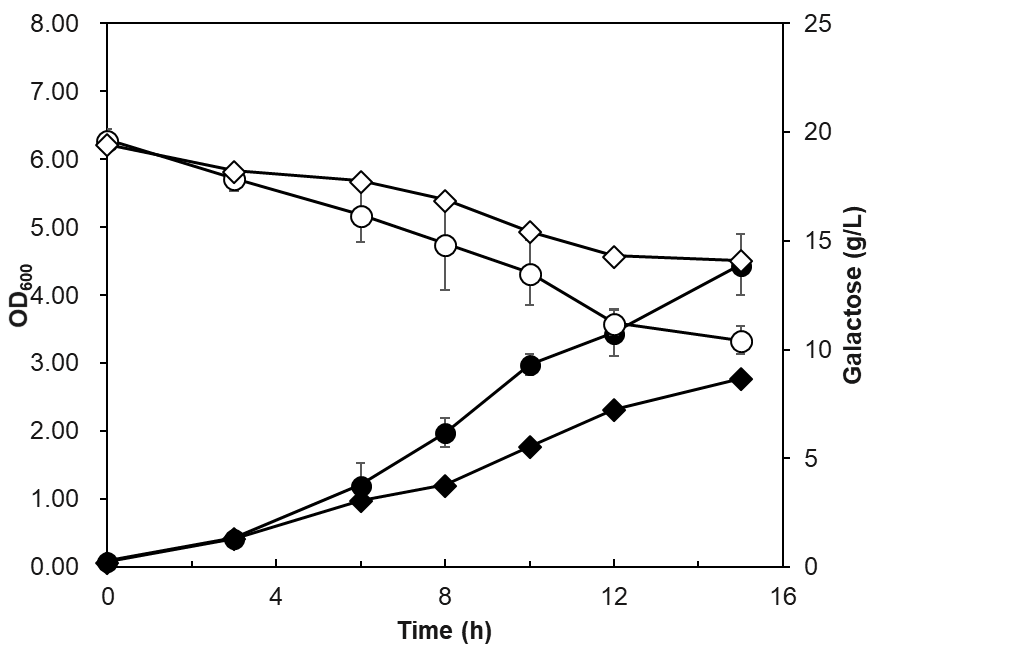


## **Figure S2.** Reverse engineering of *B. subtilis* J46 introduced mutation of *araR*^H226R^ and Δ*araR* (a) The growth profile and galactose consumption of BSGALE1 and BSGALE2 in M9 minimal medium containing 2 % (*w/v*) galactose. Black and white symbols mean optical density at 600nm and galactose concentration (g/L), respectively. Triangles and diamonds represent samples from BSGALE1 and BSGALE2 respectively. The standard deviation estimated from triplets is represented by the error bars.
